# Nomograms for Predicting Hepatocellular Carcinoma Recurrence and Overall Postoperative Patient Survival

**DOI:** 10.3389/fonc.2022.843589

**Published:** 2022-02-28

**Authors:** Lidi Ma, Kan Deng, Cheng Zhang, Haixia Li, Yingwei Luo, Yingsi Yang, Congrui Li, Xinming Li, Zhijun Geng, Chuanmiao Xie

**Affiliations:** ^1^Department of Radiology, Sun Yat-sen University Cancer Center, State Key Laboratory of Oncology in South China, Guangzhou, China; ^2^Clinical Science, Philips Healthcare, Guangzhou, China; ^3^Department of Diagnostic Radiology, Hunan Cancer Hospital, Central South University, Changsha, China; ^4^Department of Radiology, Zhujiang Hospital, Southern Medical University, Guangzhou, China

**Keywords:** hepatocellular carcinoma, nomogram, prognosis, recurrence, overall survival

## Abstract

**Background:**

Few studies have focused on the prognosis of patients with hepatocellular carcinoma (HCC) of Barcelona Clinic Liver Cancer (BCLC) stage 0‒C in terms of early recurrence and 5-years overall survival (OS). We sought to develop nomograms for predicting 5-year OS and early recurrence after curative resection of HCC, based on a clinicopathological‒radiological model. We also investigated whether different treatment methods influenced the OS of patients with early recurrence.

**Methods:**

Retrospective data, including clinical pathology, radiology, and follow-up data, were collected for 494 patients with HCC who underwent hepatectomy. Nomograms estimating OS and early recurrence were constructed using multivariate Cox regression analysis, based on the random survival forest (RSF) model. We evaluated the discrimination and calibration abilities of the nomograms using concordance indices (C-index), calibration curves, and Kaplan‒Meier curves. OS curves of different treatments for patients who had recurrence within 2 years after curative surgery were depicted and compared using the Kaplan–Meier method and the log-rank test.

**Results:**

Multivariate Cox regression revealed that BCLC stage, non-smooth margin, maximum tumor diameter, age, aspartate aminotransferase levels, microvascular invasion, and differentiation were prognostic factors for OS and were incorporated into the nomogram with good predictive performance in the training (C-index: 0.787) and testing cohorts (C-index: 0.711). A nomogram for recurrence-free survival was also developed based on four prognostic factors (BCLC stage, non-smooth margin, maximum tumor diameter, and microvascular invasion) with good predictive performance in the training (C-index: 0.717) and testing cohorts (C-index: 0.701). In comparison to the BCLC staging system, the C-index (training cohort: 0.787 vs. 0.678, 0.717 vs. 0.675; external cohort 2: 0.748 vs. 0.624, 0.729 vs. 0.587 respectively, for OS and RFS; external cohort1:0.716 vs. 0.627 for RFS, all p value<0.05), and model calibration curves all showed improved performance. Patients who underwent surgery after tumor recurrence had a higher reOS than those who underwent comprehensive treatments and supportive care.

**Conclusions:**

The nomogram, based on clinical, pathological, and radiological factors, demonstrated good accuracy in estimating OS and recurrence, which can guide follow-up and treatment of individual patients. Reoperation may be the best option for patients with recurrence in good condition.

## Introduction

Hepatocellular carcinoma (HCC) is the sixth most-common malignant neoplasm and the third leading cause of cancer-related deaths (1). HCC is currently the fifth most-common malignant tumor and the second leading cause of cancer-related death in China, with a growing mortality rate worldwide, mostly due to the prevalence of chronic hepatitis B and cirrhosis (2). Despite advances in anticancer therapies, surgical resection remains the first-line treatment and is widely performed in China. It is regarded as a curative treatment for patients with resectable HCC and is not limited to patients with very early or early liver cancer. However, 50‒70% of HCC patients develop recurrence within 5 years after surgical resection (3). The median survival in Taiwan and Japan is significantly higher than that in mainland China and Korea (4, 5). The vital cause of the poor prognosis of HCC patients in the Chinese mainland is the lack of regular surveillance. Therefore, it is important to find a robust and reliable method to predict early recurrence and postoperative survival more precisely, as this could facilitate clinical decision-making in the treatment of HCC patients, thereby further improving the prognosis of HCC patients.

Previous studies have revealed that tumor pathological characteristics, including size, number, differentiation, and vascular invasion, are significantly associated with HCC recurrence. Recently, hypointensity in the hepatobiliary phase, an incomplete capsule, and some other magnetic resonance imaging (MRI) features have been proposed as significant predictors of early recurrence and microvascular invasion (6). Some significant models have been built based on the preoperative imaging features and clinical characteristics. However, most of these studies only focused on early stage or solitary HCC and have used few combined preoperative imaging features and postoperative pathological characteristics. In addition, there have been few studies that focused on the prognosis of patients in terms of early recurrence and 5-year overall survival (OS) within the same datasets, as most study model only focused on one of these factors.

A nomogram is a simple and personalized tool based on statistical analysis and is widely used in the diagnosis and prediction of prognosis of diseases. Recently, random survival forest (RSF), a machine learning approach that combines many individual decision trees, has been applied to predict prognosis on an individual basis (7). It can deal with nonlinear effects and variable interactions easily, requiring little input from the analyst as compared to conventional linear discriminant analysis (8).

In the current study, we aimed to identify preoperative MRI features, according to the Liver Imaging Reporting and Data System (LI-RADS 2018) (9), and clinical and postoperative pathological characteristics, to establish nomograms for predicting 5-year OS and early recurrence (< 2 years) after curative resection of HCC, based on an RF model. This study also investigated whether different treatment methods influenced the OS of patients with early recurrence.

## Methods

### Patient Cohort

Our study enrolled patients with HCC after curative resection between January 2010 and December 2015 in our hospital. Basic information was also received from 86 HCC patients who were hospitalized at the Southern Medical University Zhujiang Hospital (external cohort 1) between January 2016 and January 2019. We also enrolled 52 HCC patients from Hunan cancer hospital (external cohort 2) between June 2014 and September 2016. These patient data were used for additional external validation of the proposed model. The inclusion criteria were as follows: (1) Child‒Pugh class A or B; (2) curative liver resection performed; (3) HCC diagnosis based on postoperative pathology; (4) availability of contrast-enhanced MRI scans obtained within 1 month before surgical resection; (5) a maximum diameter of the HCC lesion exceeding 10 mm. The exclusion criteria were as follows: (1) less than 24 months of follow-up; (2) use of other treatments, such as chemotherapy or interventional therapy, before surgery; (3) presence of other malignant tumors; (4) unqualified image artifacts; and (5) incomplete clinical data and follow-up information.

A total of 494 patients (428 men and 66 women; mean age, 51.28 years ± 11.29; range, 18–80 years) were enrolled in our study. For temporally independent testing, patients who underwent surgery prior to April 2015 were assigned to a training cohort (n = 346), and subsequently operated patients were assigned to a testing cohort (n = 148), at a ratio of 7:3. The training cohort was used to construct the models that were then assessed using the testing cohort.

This retrospective study was approved by the Sun Yat-sen University Cancer Center Institutional Review Board (B2021-214-01), and the requirement for obtaining informed consent was waived.

### Follow−Up and Study Endpoint

Recurrence-free survival (RFS) and OS served as the study endpoints. All patients would undergo contrast-enhanced computed tomography (CT) or MRI, and test serum alpha-fetoprotein (AFP) levels every 3 months during the first 2 years after hepatectomy, and every 6 months thereafter to determine patient survival status after surgical resection. The data were censored on December 30, 2020. RFS was defined as the time from the date of surgery to the date of first recurrence. OS was defined as the period from the time of HCC surgery to the time of death or last follow-up.

### Clinicopathological Data Collection

All patients underwent hepatectomy with tumor-margin-negative resection. Our study collected clinicopathologic characteristics that may be related to postoperative recurrence, including age, sex, history of hepatitis B, AFP, alanine aminotransferase (ALT), aspartate aminotransferase (AST), γ-glutamyl transpeptidase (GGT), total bilirubin (TBIL), albumin (ALB), platelets (PLT), Child–Pugh class (A or B), BCLC stage, liver cirrhosis, microvascular invasion (positive or negative), and pathological differentiation. Laboratory indicators, such as AFP, ALT, AST, GGT, TBIL, and ALB levels were classified into categorical variables based on a threshold value.

### MRI Data Acquisition

MRI examination was performed in our hospital using 3.0-T MRI systems (Siemens Medical Solutions, Munich, Germany; GE Medical Systems, Chicago, IL, USA; Philips Medical Systems, Best, The Netherlands) within 1 month before surgery. Our liver multiparameter MRI protocol included axial dual-echo (in-phase and opposed-phase) T1-weighted imaging, axial fat-suppressed T2-weighted imaging, diffusion-weighted imaging (DWI, b values of 0 and 800 s/mm^2^), apparent diffusion coefficient, and liver acceleration volume acquisition (LAVA-XA)-enhanced scanning. Dynamic contrast-enhanced (DCE) LAVA images were acquired at 15–20 s (arterial phase, AP), 50–55 s (portal venous phase), and 85–90 s (delayed phase) after contrast agent injection.

### Imaging Feature Evaluation

All MR images were independently reviewed by two radiologists with 3 and 10 years of experience to assess the imaging features of HCC. Discordance between the two radiologists were resolved by discussion to consensus. Radiologists were aware that the lesions were HCCs but were blinded to all other clinical data. When multiple lesions were observed, the largest lesions were analyzed. When there were more than two tumors, we defined it as 2; otherwise as 1. The imaging features were selected according to the LI-RADS 2018, including the major features [non-rim arterial phase hyperenhancement (APHE), non-peripheral washout appearance (washout), enhancing capsule appearance (capsule), size, tumor number (num)], and ancillary features (mild‒moderate T2 hyperintensity, restricted diffusion, signal homogeneity, nodule in nodule, mosaic architecture, intratumor hemorrhage, fat in mass, peritumor enhancement during mid-arterial phase, margin, etc.) (10). Our research looked at all nodules’ tumor margins to identify which had smooth margins (defined as nodular tumors in all imaging planes) and which had non-smooth margins (defined as non-nodular tumors in all imaging planes) (11, 12). The maximum cross-sectional diameter of the tumor was measured (avoiding measurement in the AP and DWI due to lesion size overestimation).

### Feature Selection and Model Construction

Eligible patients were divided into two groups: the training cohort (n = 346) and the testing cohort (n = 148) based on the time of surgery at a ratio of 7:3. We first used the RSF model to select features most relevant to the survival status of the training cohort. RSF uses a collection of decision trees to rank variables based on their importance for time-to-event, which is suitable for reducing the data dimension of highly correlated data. Subsequently, multivariate Cox regression analysis was performed using the stepwise method to identify the independent risk factors. Then, we formulated two nomograms to predict postoperative survival status based on the results of the RSF and multivariate Cox regression analysis, which can provide the clinician with a quantitative tool to predict the individual probability of OS and RFS, respectively. The concordance index (C-index), defined as the proportion of all assessable and orderly patient pairs whose predictions were line with the outcomes, was used to assess the nomogram’s discrimination abilities. To assess the nomogram’s capacity to calibrate, calibration curves were plotted. The nomogram’s clinical usefulness was demonstrated using decision curve analysis (DCA). The testing cohort was used to test and verify the model. A total risk score was calculated for each patient in the entire cohort, based on the points given for each factor in the nomogram. The patients were classified into the high-risk group and low-risk group according to the risk score using recursive partitioning. The Kaplan‒Meier method was used to compare whether the survival distributions differed between the two risk groups. OS curves of different treatments for patients who had recurrence within 2 years after curative surgery were depicted and compared using the Kaplan–Meier method and the log-rank test.

### Statistical Analysis

Statistical analysis was performed using SPSS version 22.0 software (Chicago, IL, USA) and R software (version 4.0.4). Categorical variables and continuous variables were compared between the training and testing cohorts using the chi-square test or Fisher’s exact test, and Student’s *t*-test or the Mann–Whitney U test, respectively. The RSF model analysis and Cox regression analysis were based on the Random Forest SRC and survival package, respectively. Then, stepwise regressions based on the Akaike information criterion were used to further select the above identified variables to construct multivariable Cox regression models. The nomograms were constructed to predict early recurrence and OS. The rms package was used to create the nomogram and calibration curve, while the dca R package was used to perform DCA. The function predictSurvProb() in the pec package was used to extract the survival probability predictions. The C-index was used to assess the nomograms’ prediction performance, compared by and the survival package. All reported P values were two-sided, and P  <  0.05 was considered significant, unless stated otherwise.

## Results

In total, 346 patients were included in the training cohort and 148 were included in the testing cohort. The baseline characteristics of the training and testing cohorts are presented in **Table 1**. The patient characteristics between the 2 groups were generally comparable, except that patients in the testing cohort had slightly higher percentage of negative HBsAg history, microvascular invasion, fat in mass and lower percentage of low differentiation and low PLT level. The follow-up of the training cohort is longer than the validation cohort due to the temporally independent testing. But the follow-up was over 5 years in all cases except for the event. Of the patients, 362 (73.3%) survived, while 132 (26.7%) of the patients had died by the end of the 5-year follow-up.

**Table 1:** Baseline characteristics of hepatocellular carcinoma patients.

Characteristic	Training cohort (n = 346)	Testing cohort (n = 148)	P
Sex			0.520
Male	302 (87.3)	126 (85.1)	
Female	44 (12.7)	22 (14.9)	
Age (Mean ± SD)	51.21 ± 10.95	51.45 ± 12.07	0.781
HBsAg			0.015
Positive	305 (88.2)	118 (79.7)	
Negative	41 (11.8)	30 (20.3)	
Liver cirrhosis			0.741
Present	239 (69.1)	100 (67.6)	
Absent	107 (30.9)	48 (32.4)	
Child‒Pugh class			0.674
A	341 (98.6)	147 (99.3)	
B	5 (1.4)	1 (0.7)	
BCLC stage			0.750
0	41 (11.8)	13 (8.8)	
A	238 (68.8)	106 (71.6)	
B	31 (9.0)	12 (8.1)	
C	36 (10.4)	17 (11.5)	
ALT (μ/L)			0.060
≥ 40	153 (44.2)	52 (35.1)	
< 40	193 (55.8)	96 (64.9)	
ALP (μ/L)			0.674
≥ 100	101 (29.2)	46 (31.1)	
< 100	245 (70.8)	102 (68.9)	
GGT (μ/L)			0.969
≥ 50	183 (52.9)	78 (52.7)	
< 50	163 (47.1)	70 (47.3)	
AST (μ/L)			0.892
≥ 35	159 (46.0)	69 (46.6)	
< 35	187 (54.0)	79 (53.4)	
ALB (g/L)			0.809
< 35	10 (2.9)	3 (2.0)	
≥ 35	336 (97.1)	145 (98.0)	
TBIL (μmol/L)			0.935
≥ 34	5 (1.4)	2 (1.4)	
< 34	341 (98.6)	146 (98.6)	
PT (s)			0.473
≥ 13.5	13 (3.8)	3 (2.0)	
< 13.5	333 (96.2)	145 (98.0)	
PLT (10^9^/L)			0.001
< 100	53 (15.3)	6 (4.1)	
≥ 100	293 (84.7)	142 (95.9)	
AFP (ng/ml)			0.916
≥ 200	135 (39.0)	57 (38.5)	
< 200	211 (61.0)	91 (61.5)	
Surgical resection			0.361
Anatomical	139 (40.2)	66 (44.6)	
Non-anatomical	207 (59.8)	82 (55.4)	
Differentiation (Edmondson‒Steiner)			0.001
I	21 (6.1)	4 (2.7)	
II	201 (58.1)	58 (39.2)	
II	124 (35.8)	86 (58.1)	
Microvascular invasion			0.027
Present	118 (34.1)	66 (44.6)	
Absent	228 (65.9)	82 (55.6)	
2-year recurrence (%)	135/346	49/148	0.213
Follow up (months), Median (IQR)	77 (54)	59 (12)	<0.001
Number			0.588
1	292 (84.4)	122 (82.4)	
≥ 2	54 (15.6)	26 (17.6)	
Maximum diameter (mm), Median (IQR)	41 (39)	42.5 (43)	0.342
Vascular invasion			0.893
Present	36 (10.4)	16 (10.8)	
Absent	310 (89.6)	132 (89.2)	
Nonrim APHE			0.261
Present	331 (95.7)	138 (93.2)	
Absent	15 (4.3)	10 (6.8)	
Washout			0.969
Present	327 (94.5)	140 (94.6)	
Absent	19 (5.5)	8 (5.4)	
Enhancing capsule			0.908
Present	279 (80.6)	120 (81.1)	
Absent	67 (19.4)	28 (18.9)	
Mild‒moderate T2 hyperintensity			0.297
Present	337 (97.4)	147 (99.3)	
Absent	9 (2.6)	1 (0.7)	
Restricted diffusion			0.059
Present	333 (96.2)	147 (99.3)	
Absent	13 (3.8)	1 (0.7)	
Mosaic architecture			0.316
Present	170 (49.1)	80 (54.1)	
Absent	176 (50.9)	68 (45.9)	
Nodule in nodule			0.325
Present	7 (2.0)	6 (4.1)	
Absent	339 (98.0)	142 (95.9)	
Blood product in mass			0.061
Present	47 (13.6)	30 (20.3)	
Absent	299 (86.4)	118 (79.7)	
Fat in mass			0.001
Present	6 (1.7)	22 (14.9)	
Absent	340 (98.3)	126 (85.1)	
Nodule in tumor			0.325
Present	7 (2.0)	6 (4.1)	
Absent	339 (98.0)	142 (95.9)	
Margin			0.600
Smooth	58 (16.8)	22 (14.9)	
Non-smooth	288 (83.2)	126 (85.1)	
Peritumor enhancement			0.075
Present	94 (27.2)	52 (35.1)	
Absent	252 (72.8)	96 (64.9)	
Signal homogeneity			0.366
Homogenous	114 (32.9)	55 (37.2)	
Heterogeneous	232 (67.1)	93 (62.8)	

BCLC, Barcelona Clinic Liver Cancer; AFP, alpha-fetoprotein; ALT, alanine aminotransferase; AST, aspartate aminotransferase; GGT, γ-glutamyl transpeptidase; TBIL, total bilirubin; ALB, albumin; PLT, Platelets; Nonrim APHE, non-rim arterial phase hyperenhancement.

The median OS was 77 months in the training cohort and 59 months in the testing cohort. The 1-, 2-, 3-, and 5-year OS rates were 89, 79, 77, and 73%, and 93, 88, 84, and 73% in the training and testing cohorts, respectively (P = 0.436).

The median RFS in the training and testing cohorts were 43.5 and 52 months, respectively. A total of 37.2% (184/494) of the patients had developed recurrence by the end of the 2-year follow-up. The whole mean RFS after hepatectomy was 37.39 ± 1.12 months, and the 1-, 2-, 3-, and 5-year RFS rates were 71, 62, 55, 48% and 74, 67, 59, 51% in the training and testing cohorts respectively (P = 0.160).

### Identification of Prognostic Parameters in the Training Cohort

For the 5-year OS, multivariate Cox regression analysis based on the RSF model indicated age, AST, BCLC stage, differentiation, maximum tumor diameter, microvascular invasion, and non-smooth margin involvement as independent prognostic variables. The detailed results of the multivariate analysis are shown in **Table 2**.

**Table 2:** Factors associated with overall survival after curative resection according to multivariate analysis in the training cohort.

Clinical feature	Multivariate analysis		
	HR	95% CI	P value
Age	0.977	0.9568‒0.9977	0.029
AST	1.461	0.9676‒2.5765	0.068
BCLC stage	1.461	1.1053‒1.9316	0.008
Differentiation	1.392	0.9295‒2.0856	0.109
Maximum tumor diameter	1.01	1.0025‒1.0173	0.008
Microvascular invasion	2.111	1.2629‒3.5299	0.004
Non-smooth margin	1.612	0.9648‒2.6948	0.068

HR, hazard ratio; CI, confidence interval; AST, aspartate aminotransferase; BCLC, Barcelona Clinic Liver Cancer.

For the 2-year RFS, multivariate analysis revealed that BCLC stage, maximum tumor diameter, microvascular invasion, and non-smooth margin were independent prognostic factors. The detailed results of the multivariate analysis are shown in **Table 3**.

**Table 3:** Factors associated with recurrence-free survival after curative resection according to multivariate analysis in the training cohort.

Factor	Multivariate analysis		
	HR	95% CI	P value
BCLC stage	1.429	1.127‒1.813	0.003
Maximum tumor diameter	1.01	1.004‒1.015	0.001
Microvascular invasion	1.815	1.227‒2.685	0.002
Non-smooth margin	1.765	1.147‒2.717	0.009

BCLC, Barcelona Clinic Liver Cancer; HR, hazard ratio; CI, confidence interval.

### Prognostic Nomogram for 5-Year OS and 2-Year RFS

**Figure 1:**
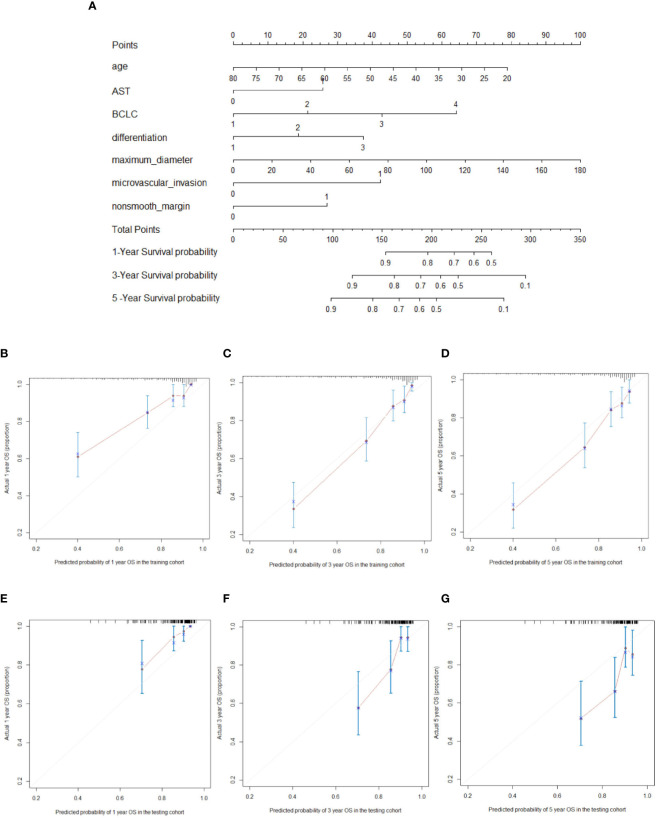
Nomogram for predicting overall survival (OS) (**A**; “1, 2, 3, 4” on the BCLC scale represent BCLC 0 A B C respectively). For every predictor, a straight vertical line upward was projected to identify the points. The total points bar was used to plot the accumulated points, and a straight vertical line yielded the 1-, 3-, and 5-year predicted post-hepatectomy survival risk. The calibration curves for predicting the 1-, 3-, and 5-year OS in the training cohort **(B–D)** and testing cohort **(E–G)** in hepatocellular carcinoma patients who underwent hepatectomy.

**Figure 2:**
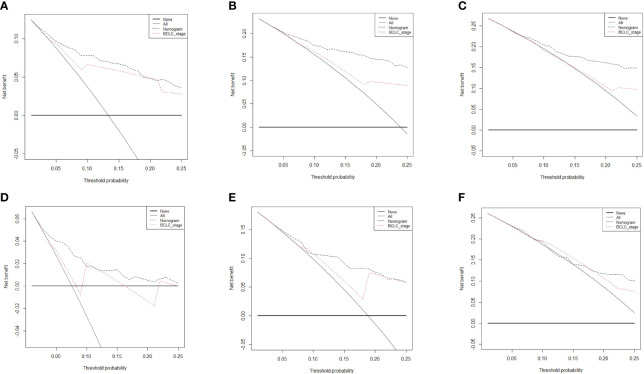
Decision curve analysis of the nomogram and Barcelona Clinic Liver Cancer system for survival prediction in hepatocellular carcinoma (HCC) patients. The 1-, 3-, and 5-year survival in the training cohort (**A–C**, respectively) and testing cohort (**D–F**, respectively) in HCC patients who underwent hepatectomy.

The prognostic nomogram for predicting 5-year OS in patients after hepatectomy is shown in **Figure 1A**. We constructed a nomogram based on the following seven independent prognostic factors: BCLC stage, non-smooth margin, maximum tumor diameter, microvascular invasion, age, AST, and differentiation. The maximum tumor diameter had the greatest impact on the OS of HCC, according to the nomogram. Users can get a precise likelihood for 1-, 3-, and 5-year survival by adding up the separate scores and calculating the total score. Higher total scores were associated with a worse prognosis. The C-index of the nomogram for predicting OS in the training cohort was 0.787 [95% confidence interval (CI), 0.726‒0.849]. The calibration curves for 1-, 3-, and 5-year OS in the training cohort presented an optimal agreement, as shown in **Figures 1B‒D**. For the testing cohort, the nomogram of predicting OS also exhibited a high accuracy, with a C-index of 0.711 (95% CI, 0.617‒0.805). The calibration curves also showed favorable calibration of the nomogram for predicting 1-, 3-, and 5-year OS in the testing cohort (**Figures 1E‒G**). DCA showed that this nomogram added benefit to predicting 5-year OS as compared with treating all patients or treating none of the patients in the training and testing cohorts (**Figures 2A‒F**).

**Figure 3:**
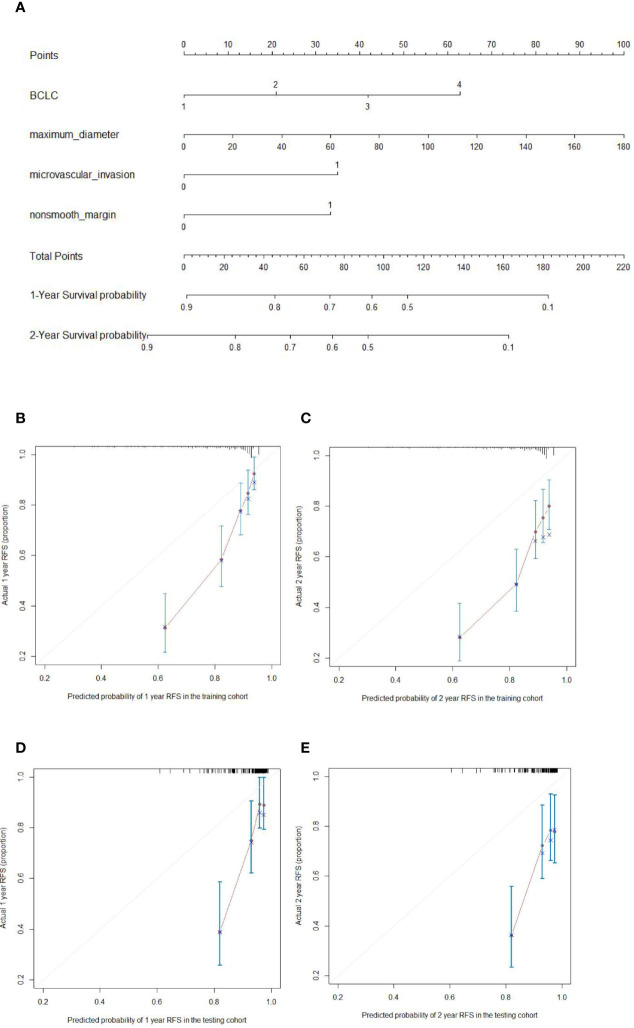
Nomogram for predicting the recurrence-free survival (RFS) (**A**; 1, 2, 3, 4 on the BCLC scale represent BCLC 0 A B C respectively) and the calibration curves for predicting the 1- and 2-year RFS in the training cohort (**B, C**, respectively) and testing cohort (**D, E**, respectively) in hepatocellular carcinoma patients who underwent hepatectomy.

**Figure 4:**
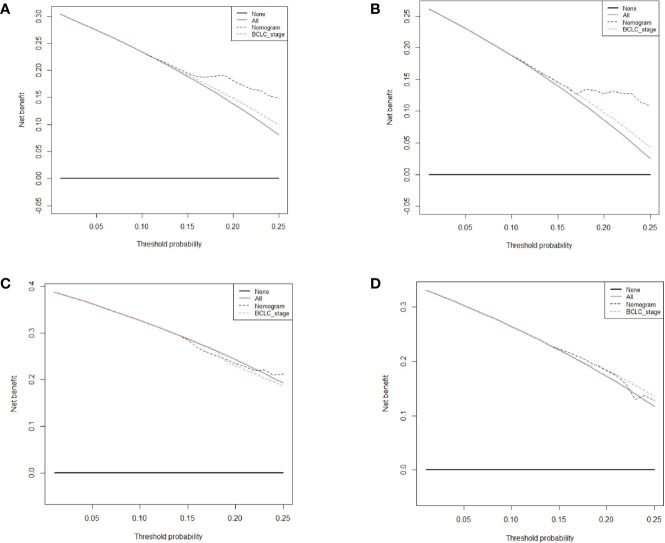
Decision curve analysis of the nomogram and Barcelona Clinic Liver Cancer system for recurrence-free survival (RFS) prediction in patients. **(A)** The 1-year RFS in the training cohort. **(B)** The 2-year RFS in the training cohort. **(C)** The 1-year RFS in the testing cohort. **(D)** The 2-year RFS in the testing cohort.

The prognostic nomogram for 2-year RFS prediction of patients after hepatectomy is shown in **Figure 3A**. The C-index of the nomogram for 2-year RFS prediction in the training cohort was 0.717 (95% CI, 0.663‒0.770). For the testing cohort, the nomogram of 2-year RFS prediction also exhibited a high accuracy with a C-index of 0.701 (95% CI, 0.616‒0.784). The calibration curves had good agreement for predicting 1- and 2-year RFS in the training and testing cohorts between the predicted and observed survival probabilities (**Figures 3B‒E**). DCA showed that using this nomogram to predict 2-year RFS was more beneficial than treating all patients or treating the non-patient scheme in the training and testing cohorts (**Figures 4A‒D**).

We further compared our model with the current BCLC staging system. The results showed that the C-index of the BCLC system was 0.67 (95%CI, 0.67‒0.72) and 0.67 (95%CI, 0.59‒0.75) for OS (**Table 4**), and 0.64 (95%CI, 0.59‒0.68) and 0.67 (95%CI, 0.60-0.74) for RFS (**Table 5**) in the training and testing cohort, respectively, which were lower than the C-index of our model (C-index = 0.787, 0.717, respectively for OS and RFS in the training cohort). The DCA for the nomogram and BCLC staging systems are shown in **Figures 2** and **4** in the training (A‒C) and testing cohorts (D‒F), respectively. The decision curve showed that using the nomogram to predict OS added more benefits than when using the BCLC staging system when the threshold probability of a patient over 5%, which was comparable in this range.

**Table 4:** C-index of the nomogram and BCLC staging system for OS.

	Training cohort			Testing cohort			External cohort2			
	C-index	95% CI	P value	C-index	95% CI	P value	C-index	95% CI	P value
Nomogram	0.787	0.726-0.849	<0.001	0.711	0.617-0.805	0.17	0.748	0.623-0.878	0.005
BCLC	0.678	0.629-0.727		0.675	0.596-0.755		0.624	0.532-0.717	

OS, overall survival; BCLC, Barcelona clinic liver cancer; CI, confidence interval.

**Table 5:** C-index of the nomogram and BCLC staging system for RFS.

	Training cohort			Testing cohort			External cohort1			External cohort 2		
	C-index	95% CI	P value	C-index	95% CI	P value	C-index	95% CI	P value	C-index	95% CI	P value
Nomogram	0.717	0.663-0.770	<0.001	0.701	0.616-0.784	0.16	0.716	0.605-0.827	0.037	0.729	0.613-0.844	0.001
BCLC	0.642	0.599-0.685		0.671	0.601-0.741		0.627	0533-0.722		0.587	0.501-0.673	

RFS, recurrence-free survival; BCLC, Barcelona clinic liver cancer; CI, confidence interval.

### External Validation of the Nomogram

**Figure 5:**
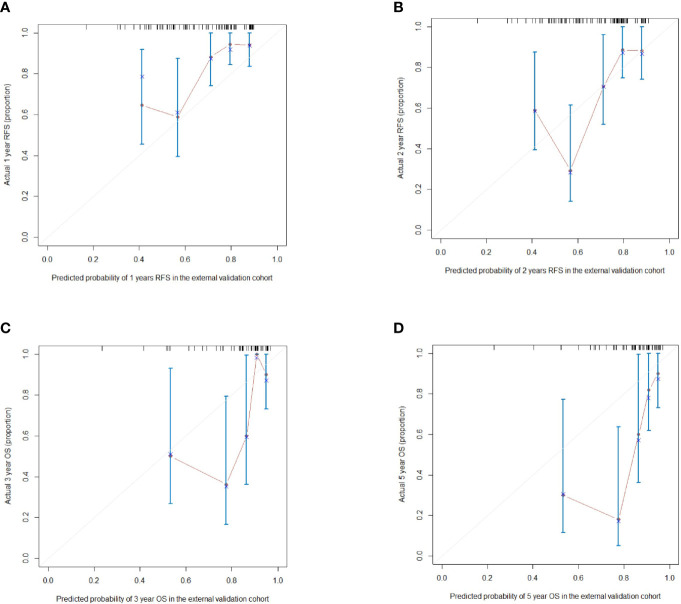
**(A, B)** The calibration curves for predicting the 1- and 2-year RFS in the external validation cohort respectively; **(C, D)** The calibration curves for predicting the 3- and 5-year OS in the external validation cohort respectively.

According to the scoring of nomogram developed with training group, the external cohort 1 was only analyzed for RFS based on follow up data, and the external cohort 2 was validated for RFS and OS. The C-index of the nomogram and BCLC system in the external cohort 1 for RFS was 0.716 (95%CI, 0.605-0.827, p=0.037), 0.627 (95%CI, 0533-0.722), respectively (**Table 5**). The C-index of the nomogram and BCLC system in the external cohort 2 for RFS was 0.729 (95%CI, 0.613-0.844, p=0.001), 0.587 (95%CI, 0.501-0.673), respectively (**Table 5**). The C-index of the nomogram and BCLC system in the external cohort 2 for OS was 0.748 (95%CI, 0.623-0.878,p=0.005), 0.624 (95%CI, 0.532-0.717), respectively (**Table 4**). Besides, the calibration curves had good agreement for predicting 1-, 2-year RFS and 3-,5-year OS in the external cohort between the predicted and observed survival probabilities (**Figure 5**).

### Prognostic Stratification Based on the Nomogram

**Figure 6:**
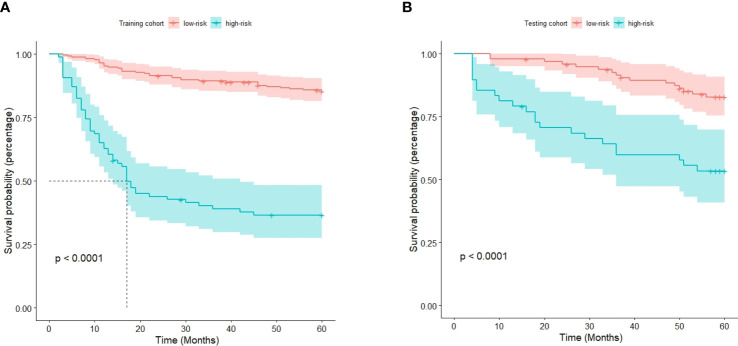
Kaplan‒Meier curves for overall survival in the training cohort **(A)** and testing cohort **(B)**.

**Figure 7:**
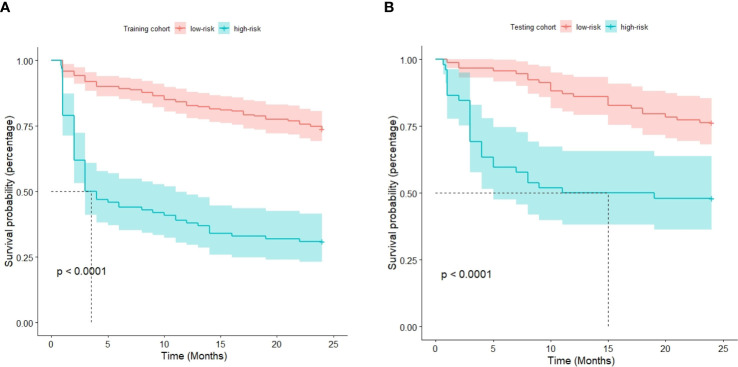
Kaplan‒Meier curves for recurrence-free survival in the training cohort **(A)** and testing cohort **(B)**.

The optimal cutoff point for the prognostic stratification classification was calculated using X-tile 3.6.1 software (https://medicine.yale.edu/lab/rimm/research/software/) in the training cohort, which were 177.7 and 83.7 for OS and RFS, respectively. Accordingly, patients with OS were divided into two subgroups: a low-risk group (total score ≤ 177.7) and a high-risk group (total score > 177.7). The Kaplan‒Meier survival analysis showed that the low-risk group was far removed from the high-risk group for OS in the training cohort, with the low-risk group demonstrating better survival than the high-risk group (**Figure 6A**). Similar results were observed in the testing cohort (**Figure 6B**). Correspondingly, patients in the present study were separated into two subgroups for RFS: a low-risk group (total score ≤ 83.7) and a high-risk group (total score > 83.7). The Kaplan‒Meier survival analyses for RFS are shown in **Figures 7A, B** for the training and testing cohorts, respectively. RFS outcomes were similar to those of OS.

### Treatment Strategy for Tumor Recurrence

186 patients (37.6%) had tumor recurrence during the 2-year follow-up after hepatectomy in the whole cohort. Of the 186 patients with tumor recurrence, 49 received supportive care, 13 underwent surgery, and 124 underwent transarterial chemoembolization (TACE)/radiotherapy/radiofrequency ablation.

**Figure 8:**
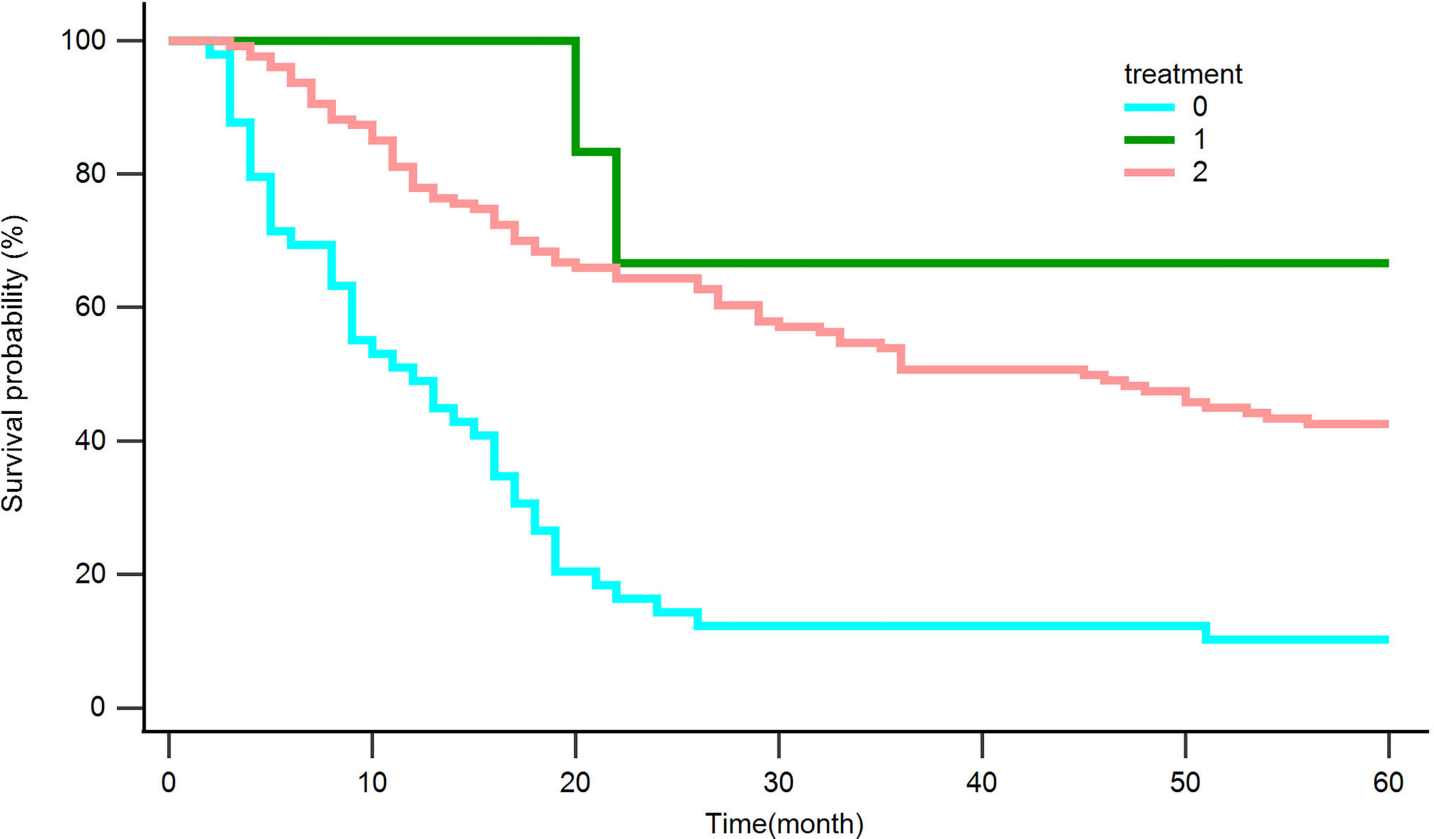
Kaplan‒Meier curves for overall survival in patients with recurrence after hepatectomy. Palliative care (0), Surgery (1), Comprehensive treatment (2).

We analyzed retreated OS (reOS), which is the time period from the date of recurrence to the date of death or the last follow-up, to see how different treatments affect recurrent tumors. Kaplan‒Meier survival analysis for reOS is shown in **Figure 8** and demonstrated that patients with hepatectomy had a higher reOS than those who received comprehensive treatments and supportive care, while patients with palliative care had the worst prognosis after tumor recurrence.

## Discussion

Interestingly, we calculated that 275 patients were candidate to liver transplant within the Milan criteria (singe lesion ≤5 cm or up to 3 lesions ≤3 cm each) in the study. However, surgical resection is still the most commonly treatment due to the severe shortage of liver donors in China. We had admitted the potential differences in the treatment of HCC between China and the West. Notably, most of the patients with BCLC stage C included in this study were due to tumor thrombus in the portal vein branches. According to the China liver cancer staging (CNLC), they are classified as stage IIIa, which the treatment methods include TACE, systemic anti-tumor therapy, surgical resection, and radiotherapy. It is possible to operate based on the standard of diagnosis and treatment of primary liver cancer in China.

Although it is the curative treatment to surgical resection for resectable HCC, the outcomes could differ between individuals due to distinct tumor biological behavior. Predicting prognosis after liver resection remains a major problem. In the present study, we established a nomogram model that combined clinical, pathological, and radiological characteristics for predicting survival outcomes in patients with HCC after resection based on RSF, a machine learning-based algorithm. The nomogram demonstrated good accuracy in estimating OS and recurrence and demonstrated guide individual patient follow-up and treatment excellently than only using BCLC staging system.

The RSF method used in this study had the potential to establish predictive models, particularly using censored survival data, and the relationship between complex response and predictor, and has been successfully used in several types of cancer (13).

In the current study, the 5-year OS rate and 2-year recurrence-free survival rate in our study were higher than others, mainly because most patients were in BCLC stage 0 or Am without other underlying diseases (14).We recognized seven and four inauspicious prognostic factors for OS and RFS with patients after hepatectomy, respectively, to establish two nomograms. BCLC stage, non-smooth margin, maximum tumor diameter, and microvascular invasion are all prognostic factors for OS and RFS. The BCLC staging system considers both tumor characteristics and liver function, including tumor extension, cancer-related symptoms, physical status, and reserved liver function (15). The BCLC staging system was positively correlated with the prognosis. Vascular invasion, the expression of the invasive biological behavior of the tumor, classified as macrovascular or microvascular invasion, is always related to a worse clinical outcome. Tumor cells infiltrating the portal vein, hepatic vein, or a major capsular vessel of the surrounding hepatic tissues is known as microvascular invasion, a histological characteristic including partial or entire lining by endothelial cells seen only under microscope (16). Our study showed that microvascular invasion is related to survival prognosis, which is consistent with several previous studies (17–20).

The maximum tumor diameter was measured mainly using radiological imaging. Several studies have reported that the maximum diameter of the tumor is an independent prognostic factor for OS and RFS (18, 19, 21, 22). Several previous studies have shown that non-smooth tumor margins are closely related to microvascular invasion and differentiation (23, 24). In recent years, Zhang et al. (25) and Chen et al. (26) discovered that non-smooth tumor margins were independent prognostic factors for OS and RFS, which was in accordance with the results of our study.

Interestingly, we found that, the younger the age, the worse the prognosis in the present study, which was inconsistent with some prior studies (27, 28). However, Giannini et al. (29) reported that older patients seem to have better prognosis than younger patients, most likely due to HCC characteristics, since older patients seem to have better pathological grades, as confirmed by Li et al. (30). Younger patients had more aggressive tumor factors than older patients, although they had better liver functional reserve (31, 32). The younger the age, the higher the AFP level. It not only seems to give rise to vascular invasion and HCC progression, but also helps to identify HCC patients who are more likely to have early recurrence and poor prognosis after surgery (33). The patients included in our study had better liver function because almost 98% of them had Child‒Pugh class A and were free of potential underlying disease and were barely affected by other factors. Although some studies have shown that pathological differentiation and AFP levels are related to prognosis, only differentiation was included in the current study. AST is measured during routine blood examinations and can reflect the grade of liver parenchymal damage. What’s more, one study showed the same result that the higher the AST level, the worse the OS (34).

Despite curative resection, HCC has a high probability of recurrence. In our subgroup analysis, 37.6% of patients had recurrence within 2 years after surgery. The optimum treatment tactic for recurrent HCC remains unclear. Although the future liver remnant is invariably smaller than other therapeutics for recurrent HCC when considering resection as a treatment modality, the OS of patients was higher than that of other treatments used in our study. Various retrospective cohort studies have demonstrated that repeat hepatectomies are secure and efficacious in patients with recurrent HCC (35). Lu et al. (36) have demonstrated that repeat hepatic resection(RHR) provides a survival benefit for recurrent HCC in comparison to RFA or TACE, particularly for patients who recurred within 2 years and those whose primary tumor burden exceeded beyond the Milan criteria. RHR offers a longer OS and RFS than RFA for patients with RHCC, which is in accordance with the current study (37).

However, this study had some limits. Firstly, of the patients, 423(85.6%) had the history of positive HBsAg. The high positive rate of HBsAg is related to the fact that HBsAg accounts for the majority of the causes of HCC in China, which means that the two nomograms can’t be used until they were validated in the West. Although it was verified by the external cohort, the sample size was relatively small. Thus, we need large-scale studies and more center clinical studies in other countries to validate our prognostic model in the future. In the current study, we only combined clinicopathological and radiological characteristics to build nomograms. Further exploration of deep learning, artificial intelligence, or genomics is required to establish better models with invisible information. In the subgroup analysis, we only compared the OS of surgical and non-surgical treatments, such as RFA and TACE, for recurrent HCC. In future, we will compare the OS of patients with recurrent HCC with various specific treatments.

In conclusion, two nomograms combining clinicopathological and multiparametric MRI data, based on the radiofrequency model demonstrated good discriminative ability in predicting postoperative 5-year OS as well as early recurrence (≤ 2 years) for HCC. For patients with recurrent HCC, the prognosis of continued surgical treatment may be better than that of non-surgical treatment.

## Data Availability Statement

The original contributions presented in the study are included in the article/**Supplementary Material**. Further inquiries can be directed to the corresponding authors.

## Ethics Statement

The studies involving human participants were reviewed and approved by Sun Yat-sen University Cancer Center Institutional Review Board (B2021-214-01). Written informed consent for participation was not required for this study in accordance with the national legislation and the institutional requirements. Written informed consent was not obtained from the individual(s) for the publication of any potentially identifiable images or data included in this article.

## Author Contributions

LM, KD, and CZ contributed to the experiment design, manuscript draft, experimental operation, and data analysis. CX, ZG and XL participated in project design, revised the manuscript, and provided technical support. LM, XL, HL, YL, YY and CL collected data. All authors contributed to the article and approved the submitted version.

## Conflict of Interest

Authors KD and HL were employed by company Philips Healthcare.

The remaining authors declare that the research was conducted in the absence of any commercial or financial relationships that could be construed as a potential conflict of interest.

## Publisher’s Note

All claims expressed in this article are solely those of the authors and do not necessarily represent those of their affiliated organizations, or those of the publisher, the editors and the reviewers. Any product that may be evaluated in this article, or claim that may be made by its manufacturer, is not guaranteed or endorsed by the publisher.

## References

[1] Sung H Ferlay J Siegel RL Laversanne M Soerjomataram I Jemal A. Global Cancer Statistics 2020: GLOBOCAN Estimates of Incidence and Mortality Worldwide for 36 Cancers in 185 Countries. CA Cancer J Clin (2021) 71:209–49. doi: 10.3322/caac.21660 33538338

[2] Cao W Chen HD Yu YW Li N Chen WQ. Changing Profiles of Cancer Burden Worldwide and in China: A Secondary Analysis of the Global Cancer Statistics 2020. Chin Med J (Engl) (2021) 134:783–91. doi: 10.1097/CM9.0000000000001474 PMC8104205 33734139

[3] Marrero JA Kulik LM Sirlin CB Zhu AX Finn RS Abecassis MM. Diagnosis, Staging, and Management of Hepatocellular Carcinoma: 2018 Practice Guidance by the American Association for the Study of Liver Diseases. Hepatology (2018) 68:723–50. doi: 10.1002/hep.29913 29624699

[4] Yang JD Hainaut P Gores GJ Amadou A Plymoth A Roberts LR. A Global View of Hepatocellular Carcinoma: Trends, Risk, Prevention and Management. Nat Rev Gastroenterol Hepatol (2019) 16:589–604. doi: 10.1038/s41575-019-0186-y 31439937 PMC6813818

[5] Park JW Chen M Colombo M Roberts LR Schwartz M Chen PJ. Global Patterns of Hepatocellular Carcinoma Management From Diagnosis to Death: The BRIDGE Study. Liver Int (2015) 35:2155–66. doi: 10.1111/liv.12818 PMC4691343 25752327

[6] Tang M Zhou Q Huang M Sun K Wu T Li X. Nomogram Development and Validation to Predict Hepatocellular Carcinoma Tumor Behavior by Preoperative Gadoxetic Acid-Enhanced MRI. Eur Radiol (2021) 31(11):8615–27. doi: 10.1007/s00330-021-07941-7 33877387

[7] Hu C Steingrimsson J. Personalized Risk Prediction in Clinical Oncology Research: Applications and Practical Issues Using Survival Trees and Random Forests. J Biopharmaceut Stat (2018) 28:333–49. doi: 10.1080/10543406.2017.1377730 PMC7196339 29048993

[8] Ishwaran H Gerds T Kogalur U Moore R Gange S Lau B. Random Survival Forests for Competing Risks. Biostat (Ox Engl) (2014) 15:757–73. doi: 10.1093/biostatistics/kxu010 PMC4173102 24728979

[9] American College of Radiology. CT/MRI LI-RADS V2018 Core. Available at: https://www.acr.org/-/media/ACR/Files/RADS/LI-RADS/LI-RADS-2018-Core.pdf?la=en.

[10] Chernyak V Fowler K Kamaya A Kielar A Elsayes K Bashir M. Liver Imaging Reporting and Data System (LI-RADS) Version 2018: Imaging of Hepatocellular Carcinoma in At-Risk Patients. Radiology (2018) 289:816–30. doi: 10.1148/radiol.2018181494 PMC6677371 30251931

[11] Ariizumi S Kitagawa K Kotera Y Takahashi Y Katagiri S Kuwatsuru R. A Non-Smooth Tumor Margin in the Hepatobiliary Phase of Gadoxetic Acid Disodium (Gd-EOB-DTPA)-Enhanced Magnetic Resonance Imaging Predicts Microscopic Portal Vein Invasion, Intrahepatic Metastasis, and Early Recurrence After Hepatectomy in Patients With Hepatocellular Carcinoma. J Hepatobil Pancreat Sci (2011) 18:575–85. doi: 10.1007/s00534-010-0369-y 21360083

[12] Chou CT Chen RC Lee CW Ko CJ Wu HK Chen YL. Prediction of Microvascular Invasion of Hepatocellular Carcinoma by Pre-Operative CT Imaging. Br J Radiol (2012) 85:778–83. doi: 10.1259/bjr/65897774 PMC3474124 21828149

[13] Zhong BY Yan ZP Sun JH Zhang L Hou ZH Zhu XL. Random Survival Forests to Predict Disease Control for Hepatocellular Carcinoma Treated With Transarterial Chemoembolization Combined With Sorafenib. Front Mol Biosci (2021) 8:618050. doi: 10.3389/fmolb.2021.618050 34095216 PMC8173079

[14] Tabrizian P Jibara G Shrager B Schwartz M Roayaie S. Recurrence of Hepatocellular Cancer After Resection: Patterns, Treatments, and Prognosis. Ann Surg (2015) 261:947–55. doi: 10.1097/SLA.0000000000000710 25010665

[15] Bruix J Sherman M American Association for the Study of Liver Diseases. Management of Hepatocellular Carcinoma: An Update. Hepatology (2011) 53:1020–2. doi: 10.1002/hep.24199 PMC3084991 21374666

[16] Zhang XP Wang K Wei XB Li LQ Sun HC Wen TF. An Eastern Hepatobiliary Surgery Hospital Microvascular Invasion Scoring System in Predicting Prognosis of Patients With Hepatocellular Carcinoma and Microvascular Invasion After R0 Liver Resection: A Large-Scale, Multicenter Study. Oncologist (2019) 24:e1476–88. doi: 10.1634/theoncologist.2018-0868 PMC6975940 31138726

[17] Kim JM Joh JW Yi NJ Choi GS Kim K Lee KW. Predicting Hepatocellular Carcinoma Recurrence Beyond Milan Criteria After Liver Resection for Solitary Hepatocellular Carcinoma. J Gastrointest Surg (2020) 24:2219–27. doi: 10.1007/s11605-019-04363-1 31482410

[18] Zhang LX Luo PQ Chen L Song DD Xu AM Xu P. Model to Predict Overall Survival in Patients With Hepatocellular Carcinoma After Curative Hepatectomy. Front Oncol (2020) 10:537526. doi: 10.3389/fonc.2020.537526 33747893 PMC7977285

[19] Pan YX Chen JC Fang AP Wang XH Chen JB Wang JC. A Nomogram Predicting the Recurrence of Hepatocellular Carcinoma in Patients After Laparoscopic Hepatectomy. Cancer Commun (Lond) (2019) 39:55. doi: 10.1186/s40880-019-0404-6 31601270 PMC6788088

[20] Shah S Cleary S Wei A Yang I Taylor B Hemming A. Recurrence After Liver Resection for Hepatocellular Carcinoma: Risk Factors, Treatment, and Outcomes. Surgery (2007) 141:330–9. doi: 10.1016/j.surg.2006.06.028 17349844

[21] Gruttadauria S Barbera F Conaldi PG Pagano D Liotta R Gringeri E. Clinical and Molecular-Based Approach in the Evaluation of Hepatocellular Carcinoma Recurrence After Radical Liver Resection. Cancers (Basel) (2021) 13(3):518. doi: 10.3390/cancers13030518 33572904 PMC7866287

[22] Chan AWH Zhong J Berhane S Toyoda H Cucchetti A Shi K. Development of Pre and Post-Operative Models to Predict Early Recurrence of Hepatocellular Carcinoma After Surgical Resection. J Hepatol (2018) 69:1284–93. doi: 10.1016/j.jhep.2018.08.027 30236834

[23] Lee S Kim SH Lee JE Sinn DH Park CK. Preoperative Gadoxetic Acid-Enhanced MRI for Predicting Microvascular Invasion in Patients With Single Hepatocellular Carcinoma. J Hepatol (2017) 67:526–34. doi: 10.1016/j.jhep.2017.04.024 28483680

[24] Renzulli M Brocchi S Cucchetti A Mazzotti F Mosconi C Sportoletti C. Can Current Preoperative Imaging Be Used to Detect Microvascular Invasion of Hepatocellular Carcinoma? Radiology (2016) 279:432–42. doi: 10.1148/radiol.2015150998 26653683

[25] Zhang Z Chen J Jiang H Wei Y Zhang X Cao L. Gadoxetic Acid-Enhanced MRI Radiomics Signature: Prediction of Clinical Outcome in Hepatocellular Carcinoma After Surgical Resection. Ann Trans Med (2020) 8:870. doi: 10.21037/atm-20-3041 PMC7396783 32793714

[26] Chen J Zhou J Kuang S Zhang Y Xie S He B. Liver Imaging Reporting and Data System Category 5: MRI Predictors of Microvascular Invasion and Recurrence After Hepatectomy for Hepatocellular Carcinoma. AJR Am J Roentgenol (2019) 213:821–30. doi: 10.2214/AJR.19.21168 31120791

[27] Tan JT Zhao C Peng NF Yang Y Zhong JH Yang T. Association Between Age and Overall Survival of Patients With Hepatocellular Carcinoma After Hepatic Resection. J Surg Oncol (2016) 114:966–70. doi: 10.1002/jso.24434 27633143

[28] Ng K Cheng N Huang J Liao M Chong C Lee K. Development and Validation of a Novel Nomogram Predicting 10-Year Actual Survival After Curative Hepatectomy for Hepatocellular Carcinoma. Surgeon (2021) 19(6):329–37. doi: 10.1016/j.hpb.2020.11.328 33423927

[29] Giannini E Romagnoli P Fasoli A Botta F Risso D Testa R. Influence of Age on Clinical Presentation, Therapeutic Options, and Prognosis in Anti-HCV Positive Cirrhotic Patients With Hepatocellular Carcinoma. Age Ageing (2002) 31:457–62. doi: 10.1093/ageing/31.6.457 12446292

[30] Li X Qi Z Du H Geng Z Li Z Qin S. Deep Convolutional Neural Network for Preoperative Prediction of Microvascular Invasion and Clinical Outcomes in Patients With HCCs. Eur Radiol (2021) 32(2):771–82. doi: 10.1007/s00330-021-08198-w 34347160

[31] Su CW Lei HJ Chau GY Hung HH Wu JC Hsia CY. The Effect of Age on the Long-Term Prognosis of Patients With Hepatocellular Carcinoma After Resection Surgery: A Propensity Score Matching Analysis. Arch Surg (2012) 147:137–44. doi: 10.1001/archsurg.2011.288 22006855

[32] Xu X Chen W Miao R Zhou Y Wang Z Zhang L. Survival Analysis of Hepatocellular Carcinoma: A Comparison Between Young Patients and Aged Patients. Chin Med J (2015) 128:1793–800. doi: 10.4103/0366-6999.159356 PMC4733704 26112722

[33] Peng SY Chen WJ Lai PL Jeng YM Sheu JC Hsu HC. High Alpha-Fetoprotein Level Correlates With High Stage, Early Recurrence and Poor Prognosis of Hepatocellular Carcinoma: Significance of Hepatitis Virus Infection, Age, P53 and Beta-Catenin Mutations. Int J Cancer (2004) 112:44–50. doi: 10.1002/ijc.20279 15305374

[34] Witjes C IJzermans J van der Eijk A Hansen B Verhoef C de Man R. And AST Are Strong Predictors for Survival After HCC Detection in Chronic HBV Patients. Netherlands J Med (2011) 69:508–13. 22279629

[35] Chan D Morris D Chua T. Clinical Efficacy and Predictors of Outcomes of Repeat Hepatectomy for Recurrent Hepatocellular Carcinoma - a Systematic Review. Surg Oncol (2013) 22:e23–30. doi: 10.1016/j.suronc.2013.02.009 23535302

[36] Lu L Mei J Kan A Ling Y Li S Wei W. Treatment Optimization for Recurrent Hepatocellular Carcinoma: Repeat Hepatic Resection Versus Radiofrequency Ablation. Cancer Med (2020) 9:2997–3005. doi: 10.1002/cam4.2951 32108433 PMC7196061

[37] Yang D Zhuang B Wang Y Xie X Xie X. Radiofrequency Ablation Versus Hepatic Resection for Recurrent Hepatocellular Carcinoma: An Updated Meta-Analysis. BMC Gastroenterol (2020) 20:402. doi: 10.1186/s12876-020-01544-0 33246417 PMC7693504

